# Supplementary material for: Gene Validation and Remodelling Using Proteogenomics of Phytophthora cinnamomi, the Causal Agent of Dieback
Source: Front Microbiol. 2021 Jul 15;12:665396. doi: 10.3389/fmicb.2021.665396 (PMC8360494; doi:10.3389/fmicb.2021.665396)
Supplement: Supplementary Material 2 — CDS coordinates of edited genes, Description: Gene coordinates of manually edited V1.0 genes. Gene identification names include the original ID from https://mycocosm.jgi.doe.gov/Phyci1 as a reference. [file Table_2.DOCX]

scaffold_1 Geneious gene 1190581 1193158 . + . estExt_fgenesh1_pm.C_10302_V2

scaffold_1 Geneious exon 1191697 1193158 . + . estExt_fgenesh1_pm.C_10302_V2

scaffold_1 Geneious exon 1190581 1190600 . + . estExt_fgenesh1_pm.C_10302_V2

scaffold_1 Geneious CDS 1190581 1190600 . + . estExt_fgenesh1_pm.C_10302_V2

scaffold_1 Geneious CDS 1191697 1193158 . + . estExt_fgenesh1_pm.C_10302_V2

scaffold_2 Geneious CDS 802177 802446 . - . CE76614_6101_V2

scaffold_2 Geneious CDS 801888 802094 . - . CE76614_6101_V2

scaffold_2 Geneious CDS 801037 801801 . - . CE76614_6101_V2

scaffold_2 Geneious CDS 800774 800947 . - . CE76614_6101_V2

scaffold_2 Geneious CDS 800619 800672 . - . CE76614_6101_V2

scaffold_2 Geneious exon 802177 802446 . - . CE76614_6101_V2

scaffold_2 Geneious exon 801888 802094 . - . CE76614_6101_V2

scaffold_2 Geneious exon 801037 801801 . - . CE76614_6101_V2

scaffold_2 Geneious exon 800774 800947 . - . CE76614_6101_V2

scaffold_2 Geneious exon 800619 800672 . - . CE76614_6101_V2

scaffold_2 Geneious gene 800619 802446 . - . CE76614_6101_V2

scaffold_5 Geneious CDS 410072 410074 . - . estExt_Genewise1Plus.C_5_t10380_V2

scaffold_5 Geneious CDS 409314 409793 . - . estExt_Genewise1Plus.C_5_t10380_V2

scaffold_5 Geneious exon 409314 409793 . - . estExt_Genewise1Plus.C_5_t10380_V2

scaffold_5 Geneious exon 410072 410074 . - . estExt_Genewise1Plus.C_5_t10380_V2

scaffold_5 Geneious gene 409314 410074 . - . estExt_Genewise1Plus.C_5_t10380_V2

scaffold_5 Geneious CDS 814040 818575 . + . e_gw1.5.959.1_V2

scaffold_5 Geneious exon 814040 818575 . + . e_gw1.5.959.1_V2

scaffold_5 Geneious gene 814040 818575 . + . e_gw1.5.959.1_V2

scaffold_10 Geneious CDS 129851 129964 . + . e_gw1.10.199.1_V2

scaffold_10 Geneious CDS 130596 130921 . + . e_gw1.10.199.1_V2

scaffold_10 Geneious CDS 131171 131321 . + . e_gw1.10.199.1_V2

scaffold_10 Geneious exon 129851 129964 . + . e_gw1.10.199.1_V2

scaffold_10 Geneious exon 130596 130921 . + . e_gw1.10.199.1_V2

scaffold_10 Geneious exon 131171 131321 . + . e_gw1.10.199.1_V2

scaffold_10 Geneious gene 129851 131321 . + . e_gw1.10.199.1_V2

scaffold_19 Geneious CDS 162308 163579 . + . e_gw1.19.375.1_V2

scaffold_19 Geneious CDS 163800 164297 . + . e_gw1.19.375.1_V2

scaffold_19 Geneious CDS 164381 164546 . + . e_gw1.19.375.1_V2

scaffold_19 Geneious exon 162308 163579 . + . e_gw1.19.375.1_V2

scaffold_19 Geneious exon 163800 164297 . + . e_gw1.19.375.1_V2

scaffold_19 Geneious exon 164381 164546 . + . e_gw1.19.375.1_V2

scaffold_19 Geneious gene 162308 164546 . + . e_gw1.19.375.1_V2

-scaffold_20 Geneious CDS 380266 380272 . - . gm1.4711_g_V2

scaffold_20 Geneious CDS 379745 380202 . - . gm1.4711_g_V2

scaffold_20 Geneious exon 379745 380202 . - . gm1.4711_g_V2

scaffold_20 Geneious exon 380266 380272 . - . gm1.4711_g_V2

scaffold_20 Geneious gene 379745 380272 . - . gm1.4711_g_V2

scaffold_20 Geneious CDS 251031 252242 . - . e_gw1.20.258.1_V2

scaffold_20 Geneious CDS 380266 380272 . - . e_gw1.20.258.1_V2

scaffold_20 Geneious exon 380266 380272 . - . e_gw1.20.258.1_V2

scaffold_20 Geneious exon 251031 252242 . - . e_gw1.20.258.1_V2

scaffold_20 Geneious gene 251031 252242 . - . e_gw1.20.258.1_V2

scaffold_21 Geneious CDS 370422 371894 . - . e_gw1.21.210.1_V2

scaffold_21 Geneious CDS 369876 370080 . - . e_gw1.21.210.1_V2

scaffold_21 Geneious CDS 369033 369793 . - . e_gw1.21.210.1_V2

scaffold_21 Geneious exon 370422 371894 . - . e_gw1.21.210.1_V2

scaffold_21 Geneious gene 369033 371894 . - . e_gw1.21.210.1_V2

scaffold_21 Geneious exon 369033 369793 . - . e_gw1.21.210.1_V2

scaffold_21 Geneious exon 369876 370080 . - . e_gw1.21.210.1_V2

scaffold_23 Geneious CDS 374374 374862 . - . fgenesh1_kg.23_#_87_V2

scaffold_23 Geneious exon 374374 374862 . - . fgenesh1_kg.23_#_87_V2

scaffold_23 Geneious gene 374374 374862 . - . fgenesh1_kg.23_#_87_V2

scaffold_25 Geneious CDS 360286 360963 . - . fgenesh1_kg.25_#_42_V2

scaffold_25 Geneious exon 360286 360963 . - . fgenesh1_kg.25_#_42_V2

scaffold_25 Geneious gene 360286 360963 . - . fgenesh1_kg.25_#_42_V2

scaffold_28 Geneious gene 68133 69671 . + . e_gw1.28.366.1_V2

scaffold_28 Geneious CDS 68133 69671 . + . e_gw1.28.366.1_V2

scaffold_28 Geneious exon 68133 69671 . + . e_gw1.28.366.1_V2

scaffold_32 Geneious CDS 338764 340050 . + . fgenesh1_pm.32_#_44_V2

scaffold_32 Geneious CDS 340132 340213 . + . fgenesh1_pm.32_#_44_V2

scaffold_32 Geneious CDS 341031 341145 . + . fgenesh1_pm.32_#_44_V2

scaffold_32 Geneious CDS 342542 342651 . + . fgenesh1_pm.32_#_44_V2

scaffold_32 Geneious CDS 342748 344672 . + . fgenesh1_pm.32_#_44_V2

scaffold_32 Geneious exon 338764 340050 . + . fgenesh1_pm.32_#_44_V2

scaffold_32 Geneious exon 340132 340213 . + . fgenesh1_pm.32_#_44_V2

scaffold_32 Geneious exon 341031 341145 . + . fgenesh1_pm.32_#_44_V2

scaffold_32 Geneious exon 342542 342651 . + . fgenesh1_pm.32_#_44_V2

scaffold_32 Geneious exon 342748 344672 . + . fgenesh1_pm.32_#_44_V2

scaffold_32 Geneious gene 338764 344672 . + . fgenesh1_pm.32_#_44_V2

scaffold_32 Geneious CDS 342542 342651 . + . fgenesh1_kg.38_#_4_V2

scaffold_32 Geneious CDS 342748 344672 . + . fgenesh1_kg.38_#_4_V2

scaffold_32 Geneious exon 338764 340050 . + . fgenesh1_kg.38_#_4_V2

scaffold_38 Geneious CDS 57194 57709 . - . fgenesh1_kg.38_#_4_V2

scaffold_38 Geneious exon 57194 57709 . - . fgenesh1_kg.38_#_4_V2

scaffold_38 Geneious gene 57194 57709 . - . fgenesh1_kg.38_#_4_V2

scaffold_44 Geneious CDS 8990 9119 . + . gw1.44.72.1_V2

scaffold_44 Geneious CDS 9203 10053 . + . gw1.44.72.1_V2

scaffold_44 Geneious exon 8990 9119 . + . gw1.44.72.1_V2

scaffold_44 Geneious exon 9203 10053 . + . gw1.44.72.1_V2

scaffold_44 Geneious gene 8990 10053 . + . gw1.44.72.1_V2

scaffold_62 Geneious CDS 121156 121185 . + . fgenesh1_pg.62_#_28_V2

scaffold_62 Geneious CDS 121477 123099 . + . fgenesh1_pg.62_#_28_V2

scaffold_62 Geneious CDS 123263 123308 . + . fgenesh1_pg.62_#_28_V2

scaffold_62 Geneious CDS 123561 123601 . + . fgenesh1_pg.62_#_28_V2

scaffold_62 Geneious CDS 123662 123926 . + . fgenesh1_pg.62_#_28_V2

scaffold_62 Geneious CDS 123950 127406 . + . fgenesh1_pg.62_#_28_V2

scaffold_62 Geneious CDS 127491 127860 . + . fgenesh1_pg.62_#_28_V2

scaffold_62 Geneious exon 121156 121185 . + . fgenesh1_pg.62_#_28_V2

scaffold_62 Geneious exon 121477 123099 . + . fgenesh1_pg.62_#_28_V2

scaffold_62 Geneious exon 123263 123308 . + . fgenesh1_pg.62_#_28_V2

scaffold_62 Geneious exon 123561 123601 . + . fgenesh1_pg.62_#_28_V2

scaffold_62 Geneious exon 123662 123926 . + . fgenesh1_pg.62_#_28_V2

scaffold_62 Geneious exon 123950 127406 . + . fgenesh1_pg.62_#_28_V2

scaffold_62 Geneious exon 127491 127860 . + . fgenesh1_pg.62_#_28_V2

scaffold_62 Geneious gene 121156 127860 . + . fgenesh1_pg.62_#_28_V2

scaffold_64 Geneious CDS 116702 117700 . + . estExt_fgenesh1_pg.C_640028_V2

scaffold_64 Geneious exon 116702 117700 . + . estExt_fgenesh1_pg.C_640028_V2

scaffold_64 Geneious gene 116702 117700 . + . estExt_fgenesh1_pg.C_640028_V2

scaffold_65 Geneious gene 167297 169246 . + . gw1.65.13.1_V2

scaffold_65 Geneious CDS 167297 169246 . + . gw1.65.13.1_V2

scaffold_65 Geneious exon 167297 169246 . + . gw1.65.13.1_V2

scaffold_66 Geneious CDS 234972 236015 . - . e_gw1.66.78.1_V2

scaffold_66 Geneious CDS 232334 233116 . - . e_gw1.66.78.1_V2

scaffold_66 Geneious CDS 232145 232333 . - . e_gw1.66.78.1_V2

scaffold_66 Geneious exon 234972 236015 . - . e_gw1.66.78.1_V2

scaffold_66 Geneious exon 232334 233116 . - . e_gw1.66.78.1_V2

scaffold_66 Geneious exon 232145 232333 . - . e_gw1.66.78.1_V2

scaffold_66 Geneious gene 232145 236015 . - . e_gw1.66.78.1_V2

scaffold_68 Geneious CDS 229907 230146 . - . estExt_Genewise1.C_680214_V2

scaffold_68 Geneious CDS 228752 229513 . - . estExt_Genewise1.C_680214_V2

scaffold_68 Geneious exon 229907 230146 . - . estExt_Genewise1.C_680214_V2

scaffold_68 Geneious exon 228752 229513 . - . estExt_Genewise1.C_680214_V2

scaffold_68 Geneious gene 228752 230146 . - . estExt_Genewise1.C_680214_V2

scaffold_75 Geneious CDS 43818 44102 . + . e_gw1.75.179.1_V2

scaffold_75 Geneious CDS 44176 44471 . + . e_gw1.75.179.1_V2

scaffold_75 Geneious CDS 44555 45079 . + . e_gw1.75.179.1_V2

scaffold_75 Geneious CDS 45147 45693 . + . e_gw1.75.179.1_V2

scaffold_75 Geneious exon 43818 44102 . + . e_gw1.75.179.1_V2

scaffold_75 Geneious exon 44176 44471 . + . e_gw1.75.179.1_V2

scaffold_75 Geneious exon 44555 45079 . + . e_gw1.75.179.1_V2

scaffold_75 Geneious exon 45147 45693 . + . e_gw1.75.179.1_V2

scaffold_75 Geneious gene 43818 45693 . + . e_gw1.75.179.1_V2

scaffold_79 Geneious CDS 105136 110853 . - . fgenesh1_kg.79_V2

scaffold_79 Geneious exon 105136 110853 . - . fgenesh1_kg.79_V2

scaffold_79 Geneious gene 105136 110853 . - . fgenesh1_kg.79_V2

scaffold_80 Geneious CDS 100981 102753 . - . e_gw1.80.224.1_V2

scaffold_80 Geneious exon 100981 102753 . - . e_gw1.80.224.1_V2

scaffold_80 Geneious gene 100981 102753 . - . e_gw1.80.224.1_V2

scaffold_84 Geneious CDS 89590 89678 . - . estExt_Genewise1.C_840092_V2

scaffold_84 Geneious CDS 88294 89457 . - . estExt_Genewise1.C_840092_V2

scaffold_84 Geneious exon 89590 89678 . - . estExt_Genewise1.C_840092_V2

scaffold_84 Geneious exon 88294 89457 . - . estExt_Genewise1.C_840092_V2

scaffold_84 Geneious gene 88294 89678 . - . estExt_Genewise1.C_840092_V2

scaffold_89 Geneious CDS 199637 200500 . + . fgenesh1_kg.89_#_46_V2

scaffold_89 Geneious exon 199637 200500 . + . fgenesh1_kg.89_#_46_V2

scaffold_89 Geneious gene 199637 200500 . + . fgenesh1_kg.89_#_46_V2

scaffold_95 Geneious CDS 71841 73105 . + . e_gw1.95.148.1_exon1_2_V2

scaffold_95 Geneious CDS 73216 73384 . + . e_gw1.95.148.1_exon1_2_V2

scaffold_95 Geneious exon 71841 73105 . + . e_gw1.95.148.1_V2

scaffold_95 Geneious exon 73216 73384 . + . e_gw1.95.148.1_V2

scaffold_95 Geneious gene 71841 73384 . + . e_gw1.95.148.1_V2

scaffold_97 Geneious CDS 137445 138728 . + . fgenesh1_kg.97_#_27_V2

scaffold_97 Geneious exon 137445 138728 . + . fgenesh1_kg.97_#_27_V2

scaffold_97 Geneious gene 137445 138728 . + . fgenesh1_kg.97_#_27_V2

scaffold_108 Geneious exon 125835 125996 . - . estExt_Genewise1Plus.C_1080115_V2

scaffold_108 Geneious exon 120154 121185 . - . estExt_Genewise1Plus.C_1080115_V2

scaffold_108 Geneious exon 119728 120117 . - . estExt_Genewise1Plus.C_1080115_V2

scaffold_108 Geneious CDS 125835 125996 . - . estExt_Genewise1Plus.C_1080115_V2

scaffold_108 Geneious CDS 120154 121185 . - . estExt_Genewise1Plus.C_1080115_V2

scaffold_108 Geneious CDS 119728 120117 . - . estExt_Genewise1Plus.C_1080115_V2

scaffold_108 Geneious gene 119728 125996 . - . estExt_Genewise1Plus.C_1080115_V2

scaffold_144 Geneious CDS 161366 161375 . + . fgenesh1_kg.144_#_31_V2

scaffold_144 Geneious CDS 161430 161504 . + . fgenesh1_kg.144_#_31_V2

scaffold_144 Geneious CDS 161535 161660 . + . fgenesh1_kg.144_#_31_V2

scaffold_144 Geneious CDS 161875 162065 . + . fgenesh1_kg.144_#_31_V2

scaffold_144 Geneious CDS 162240 162314 . + . fgenesh1_kg.144_#_31_V2

scaffold_144 Geneious CDS 162379 162687 . + . fgenesh1_kg.144_#_31_V2

scaffold_144 Geneious exon 161366 161375 . + . fgenesh1_kg.144_#_31_V2

scaffold_144 Geneious exon 161430 161504 . + . fgenesh1_kg.144_#_31_V2

scaffold_144 Geneious exon 161535 161660 . + . fgenesh1_kg.144_#_31_V2

scaffold_144 Geneious exon 161875 162065 . + . fgenesh1_kg.144_#_31_V2

scaffold_144 Geneious exon 162240 162314 . + . fgenesh1_kg.144_#_31_V2

scaffold_144 Geneious exon 162379 162687 . + . fgenesh1_kg.144_#_31_V2

scaffold_144 Geneious gene 161366 162687 . + . fgenesh1_kg.144_#_31_V2

scaffold_148 Geneious CDS 164447 167677 . + . gm1.15414_g_V2

scaffold_148 Geneious exon 164447 167677 . + . gm1.15414_g_V2

scaffold_148 Geneious gene 164447 167677 . + . gm1.15414_g_V2

scaffold_160 Geneious CDS 61138 61881 . + . gw1.160.19.1_V2

scaffold_160 Geneious exon 61138 61881 . + . gw1.160.19.1_V2

scaffold_160 Geneious gene 61138 61881 . + . gw1.160.19.1_V2

scaffold_179 Geneious CDS 39992 40036 . + . fgenesh1_pm.179_#_14_V2

scaffold_179 Geneious CDS 40076 40101 . + . fgenesh1_pm.179_#_14_V2

scaffold_179 Geneious CDS 40204 40927 . + . fgenesh1_pm.179_#_14_V2

scaffold_179 Geneious CDS 42325 42399 . + . fgenesh1_pm.179_#_14_V2

scaffold_179 Geneious exon 39992 40036 . + . fgenesh1_pm.179_#_14_V2

scaffold_179 Geneious exon 40076 40101 . + . fgenesh1_pm.179_#_14_V2

scaffold_179 Geneious exon 40204 40927 . + . fgenesh1_pm.179_#_14_V2

scaffold_179 Geneious exon 42325 42399 . + . fgenesh1_pm.179_#_14_V2

scaffold_179 Geneious gene 39992 42399 . + . fgenesh1_pm.179_#_14_V2

scaffold_179 Geneious CDS 62881 64227 . - . e_gw1.179.22.1_V2

scaffold_179 Geneious exon 62881 64227 . - . e_gw1.179.22.1_V2

scaffold_179 Geneious gene 62881 64227 . - . e_gw1.179.22.1_V2

scaffold_179 Geneious gene 73259 74149 . + . e_gw1.179.10.1_V2

scaffold_179 Geneious CDS 73259 74149 . + . e_gw1.179.10.1_V2

scaffold_179 Geneious exon 73259 74149 . + . e_gw1.179.10.1_V2

scaffold_193 Geneious exon 17646 20819 . + . gw1.193.42.1_V2

scaffold_193 Geneious CDS 17646 20819 . + . gw1.193.42.1_V2

scaffold_193 Geneious gene 17646 20819 . + . gw1.193.42.1_V2

scaffold_193 Geneious CDS 64264 64410 . - . CE70043_1777_V2

scaffold_193 Geneious CDS 64110 64193 . - . CE70043_1777_V2

scaffold_193 Geneious CDS 63956 64036 . - . CE70043_1777_V2

scaffold_193 Geneious CDS 63240 63887 . - . CE70043_1777_V2

scaffold_193 Geneious CDS 63095 63163 . - . CE70043_1777_V2

scaffold_193 Geneious CDS 62770 63015 . - . CE70043_1777_V2

scaffold_193 Geneious exon 64264 64410 . - . CE70043_1777_V2

scaffold_193 Geneious exon 64110 64193 . - . CE70043_1777_V2

scaffold_193 Geneious exon 63956 64036 . - . CE70043_1777_V2

scaffold_193 Geneious exon 63240 63887 . - . CE70043_1777_V2

scaffold_193 Geneious exon 63095 63163 . - . CE70043_1777_V2

scaffold_193 Geneious exon 62770 63015 . - . CE70043_1777_V2

scaffold_193 Geneious gene 62770 64410 . - . CE70043_1777_V2

scaffold_277 Geneious CDS 23968 24096 . + . fgenesh1_kg.277_#_5_V2

scaffold_277 Geneious CDS 24212 25114 . + . fgenesh1_kg.277_#_5_V2

scaffold_277 Geneious exon 23968 24096 . + . fgenesh1_kg.277_#_5_V2

scaffold_277 Geneious exon 24212 25114 . + . fgenesh1_kg.277_#_5_V2

scaffold_277 Geneious gene 23968 25114 . + . fgenesh1_kg.277_#_5_V2

scaffold_451 Geneious CDS 1423 2856 . + . estExt_fgenesh1_pm.C_4510002_V2

scaffold_451 Geneious CDS 4666 4704 . + . estExt_fgenesh1_pm.C_4510002_V2

scaffold_451 Geneious exon 1423 2856 . + . estExt_fgenesh1_pm.C_4510002_V2

scaffold_451 Geneious exon 4666 4704 . + . estExt_fgenesh1_pm.C_4510002_V2

scaffold_451 Geneious gene 1423 4704 . + . estExt_fgenesh1_pm.C_4510002_V2

scaffold_432 Geneious CDS 4946 6094 . - . fgenesh1_pg.432_#_2

scaffold_432 Geneious CDS 2903 3931 . - . fgenesh1_pg.432_#_2

scaffold_432 Geneious exon 4946 6094 . - . fgenesh1_pg.432_#_2

scaffold_432 Geneious exon 2903 3931 . - . fgenesh1_pg.432_#_2

scaffold_432 Geneious gene 2903 6094 . - . fgenesh1_pg.432_#_2

scaffold_382 Geneious gene 16891 17427 . + . gm1.21101_g_V2

scaffold_382 Geneious exon 16891 17427 . + . gm1.21101_g_V2

scaffold_382 Geneious CDS 16891 17427 . + . gm1.21101_g_V2

scaffold_318 Geneious CDS 49186 50469 . - . estExt_Genewise1.C_3180059_V2

scaffold_318 Geneious CDS 46594 49005 . - . estExt_Genewise1.C_3180059_V2

scaffold_318 Geneious exon 49186 50469 . - . estExt_Genewise1.C_3180059_V2

scaffold_318 Geneious exon 46594 49005 . - . estExt_Genewise1.C_3180059_V2

scaffold_318 Geneious gene 46594 50469 . - . estExt_Genewise1.C_3180059_V2

scaffold_295 Geneious CDS 10546 10803 . + . fgenesh1_kg.295.28.1_V2

scaffold_295 Geneious CDS 10860 11462 . + . fgenesh1_kg.295.28.1_V2

scaffold_295 Geneious exon 10546 10803 . + . fgenesh1_kg.295.28.1_V2

scaffold_295 Geneious gene 10546 11462 . + . fgenesh1_kg.295.28.1_V2

scaffold_295 Geneious exon 10860 11462 . + . fgenesh1_kg.295.28.1_V2

scaffold_32 Geneious CDS 210980 212173 . - . e_gw1.32.257.1_V2

scaffold_32 Geneious exon 210980 212173 . - . e_gw1.32.257.1_V2

scaffold_32 Geneious gene 210980 212173 . - . e_gw1.32.257.1_V2

scaffold_204 Geneious CDS 40454 41710 . + . fgenesh1_kg.204_#_6_#_Locus963v1rpkm260.20_V2

scaffold_204 Geneious exon 40454 41710 . + . fgenesh1_kg.204_#_6_#_Locus963v1rpkm260.20_V2

scaffold_204 Geneious gene 40454 41710 . + . fgenesh1_kg.204_#_6_#_Locus963v1rpkm260.20_V2

scaffold_25 Geneious CDS 100226 101500 . - . e_gw1.25.388.1_V2

scaffold_25 Geneious exon 100226 101500 . - . e_gw1.25.388.1_V2

scaffold_25 Geneious gene 100226 101500 . - . e_gw1.25.388.1_V2

scaffold_54 Geneious exon 174487 174576 . - . fgenesh1_pm.54_#_29_V2

scaffold_54 Geneious exon 173203 174327 . - . fgenesh1_pm.54_#_29_V2

scaffold_54 Geneious exon 172379 173135 . - . fgenesh1_pm.54_#_29_V2

scaffold_54 Geneious exon 172225 172308 . - . fgenesh1_pm.54_#_29_V2

scaffold_54 Geneious gene 172225 174576 . - . fgenesh1_pm.54_#_29_V2

scaffold_54 Geneious CDS 174487 174576 . - . fgenesh1_pm.54_#_29_V2

scaffold_54 Geneious CDS 173203 174327 . - . fgenesh1_pm.54_#_29_V2

scaffold_54 Geneious CDS 172379 173135 . - . fgenesh1_pm.54_#_29_V2

scaffold_54 Geneious CDS 172225 172308 . - . fgenesh1_pm.54_#_29_V2

scaffold_87 Geneious CDS 128124 129590 . - . genesh1_kg.87_#_21_V2

scaffold_87 Geneious exon 128124 129590 . - . genesh1_kg.87_#_21_V2

scaffold_87 Geneious gene 128124 129590 . - . genesh1_kg.87_#_21_V2

scaffold_74 Geneious CDS 114133 117912 . - . CE195689_14338_V2

scaffold_74 Geneious exon 114133 117912 . - . CE195689_14338_V2

scaffold_74 Geneious gene 114133 117912 . - . CE195689_14338_V2

scaffold_102 Geneious gene 207461 211954 . - . estExt_Genewise1Plus.C_1020136_V2

scaffold_102 Geneious CDS 210112 211954 . - . estExt_Genewise1Plus.C_1020136_V2

scaffold_102 Geneious CDS 207463 207716 . - . estExt_Genewise1Plus.C_1020136_V2

scaffold_102 Geneious exon 210112 211954 . - . estExt_Genewise1Plus.C_1020136_V2

scaffold_102 Geneious exon 207463 207716 . - . estExt_Genewise1Plus.C_1020136_V2

scaffold_9 Geneious CDS 34262 42919 . + . estExt_fgenesh1_pm.C_90019_fgenesh1_pm.9_#_20_e_gw1.9.526.1_V2

scaffold_9 Geneious exon 34262 42919 . + . estExt_fgenesh1_pm.C_90019_fgenesh1_pm.9_#_20_e_gw1.9.526.1_V2

scaffold_9 Geneious gene 34262 42919 . + . estExt_fgenesh1_pm.C_90019_fgenesh1_pm.9_#_20_e_gw1.9.526.1_V2

scaffold_40 Geneious CDS 215151 215702 . - . estExt_Genewise1Plus.C_400155_V2

scaffold_40 Geneious CDS 210462 211175 . - . estExt_Genewise1Plus.C_400155_V2

scaffold_40 Geneious exon 215151 215702 . - . estExt_Genewise1Plus.C_400155_V2

scaffold_40 Geneious exon 210462 211175 . - . estExt_Genewise1Plus.C_400155_V2

scaffold_40 Geneious gene 210462 215702 . - . estExt_Genewise1Plus.C_400155_V2

scaffold_47 Geneious CDS 248 2893 . - . estExt_fgenesh1_pm.C_470001_V2

scaffold_47 Geneious exon 248 2893 . - . estExt_fgenesh1_pm.C_470001_V2

scaffold_47 Geneious gene 248 2893 . - . estExt_fgenesh1_pm.C_470001_V2

scaffold_47 Geneious CDS 286994 290311 . - . e_gw1.47.245.1_V2

scaffold_47 Geneious exon 286994 290311 . - . e_gw1.47.245.1_V2

scaffold_47 Geneious gene 286994 290311 . - . e_gw1.47.245.1_V2

scaffold_48 Geneious CDS 282504 283122 . + . e_gw1.48.356.1_V2

scaffold_48 Geneious CDS 283357 283529 . + . e_gw1.48.356.1_V2

scaffold_48 Geneious exon 282504 283122 . + . e_gw1.48.356.1_V2

scaffold_48 Geneious exon 283357 283529 . + . e_gw1.48.356.1_V2

scaffold_48 Geneious gene 282504 283529 . + . e_gw1.48.356.1_V2

scaffold_49 Geneious CDS 211448 211681 . + . e_gw1.49.253.1_V2

scaffold_49 Geneious CDS 211771 211959 . + . e_gw1.49.253.1_V2

scaffold_49 Geneious CDS 212060 213319 . + . e_gw1.49.253.1_V2

scaffold_49 Geneious exon 211448 211681 . + . e_gw1.49.253.1_V2

scaffold_49 Geneious exon 211771 211959 . + . e_gw1.49.253.1_V2

scaffold_49 Geneious exon 212060 213319 . + . e_gw1.49.253.1_V2

scaffold_49 Geneious gene 211448 213319 . + . e_gw1.49.253.1_V2

scaffold_100 Geneious CDS 172670 175243 . - . estExt_Genewise1.C_1000146_V2

scaffold_100 Geneious CDS 169697 172621 . - . estExt_Genewise1.C_1000146_V2

scaffold_100 Geneious CDS 167231 169639 . - . estExt_Genewise1.C_1000146_V2

scaffold_100 Geneious CDS 167012 167155 . - . estExt_Genewise1.C_1000146_V2

scaffold_100 Geneious exon 172670 175243 . - . estExt_Genewise1.C_1000146_V2

scaffold_100 Geneious exon 169697 172621 . - . estExt_Genewise1.C_1000146_V2

scaffold_100 Geneious exon 167231 169639 . - . estExt_Genewise1.C_1000146_V2

scaffold_100 Geneious exon 167012 167155 . - . estExt_Genewise1.C_1000146_V2

scaffold_100 Geneious gene 167012 175243 . - . estExt_Genewise1.C_1000146_V2

scaffold_108 Geneious CDS 144024 146360 . - . e_gw1.108.58.1V2

scaffold_108 Geneious exon 144024 146360 . - . e_gw1.108.58.1_V2

scaffold_108 Geneious gene 144024 146360 . - . e_gw1.108.58.1_V2

scaffold_199 Geneious exon 73065 73237 . + . fgenesh1_pg.199_#_16_V2

scaffold_199 Geneious exon 73618 75732 . + . fgenesh1_pg.199_#_16_V2

scaffold_199 Geneious exon 75814 76125 . + . fgenesh1_pg.199_#_16_V2

scaffold_199 Geneious gene 72954 76125 . + . fgenesh1_pg.199_#_16_V2

scaffold_199 Geneious CDS 73438 75732 . + . fgenesh1_pg.199_#_16_V2

scaffold_199 Geneious CDS 75814 76125 . + . fgenesh1_pg.199_#_16_V2

scaffold_199 Geneious CDS 73065 73237 . + . fgenesh1_pg.199_#_16_V2

scaffold_199 Geneious exon 72954 72956 . + . fgenesh1_pg.199_#_16_V2

scaffold_199 Geneious CDS 72954 72956 . + . fgenesh1_pg.199_#_16_V2

scaffold_243 Geneious CDS 2849 7276 . - . gw1.243.65.1, gw1.243.79.1_V2

scaffold_243 Geneious exon 2849 7276 . - . gw1.243.65.1, gw1.243.79.1_V2

scaffold_243 Geneious gene 2849 7276 . - . gw1.243.65.1, gw1.243.79.1_V2

scaffold_243 Geneious CDS 19913 24325 . + . e_gw1.243.97.1_V2

scaffold_243 Geneious exon 19913 24325 . + . e_gw1.243.97.1_V2

scaffold_243 Geneious gene 19913 24325 . + . e_gw1.243.97.1_V2
